# Comparative Study on Online Prediction of TP2 Rolled Copper Tube Wall Thickness Based on Different Proxy Models

**DOI:** 10.3390/ma17235685

**Published:** 2024-11-21

**Authors:** Fengli Yue, Zhuo Sha, Hongyun Sun, Huan Liu, Dayong Chen, Jinsong Liu, Chuanlai Chen

**Affiliations:** 1 Automotive and Transportation College, Shenyang Ligong University, Shenyang 110159, China; zhuosha0820@163.com (Z.S.); sunhy5006@163.com (H.S.); liuh@sylu.edu.cn (H.L.); jsliu@imr.ac.cn (J.L.); 2 Shi Changxu Materials Innovation Center, Institute of Metal Research, Chinese Academy of Sciences, Shenyang 110016, China; dychen15b@imr.ac.cn; 3 Changzhou Enreach Copper Co., Ltd., Changzhou 213149, China; slgcdy@163.com

**Keywords:** joint drawing, ultrasonic testing, numerical simulation, neural network

## Abstract

The wall thickness of the TP2 copper tube casting billet is not uniform after a three-roll planetary rotational rolling, which affects the wall thickness uniformity of the copper tube in the subsequent process. In order to study the influence of wall thickness at different positions of copper pipe after rolling on the wall thickness of copper pipe after joint drawing, an online ultrasonic test platform was used to measure the wall thickness of copper pipe after tying, and based on the test data, a finite element model of copper pipe billet was established, and the numerical simulation of joint drawing wall thickness was conducted. Based on the results of the ultrasonic testing experiment and finite element simulation, different neural network models were used to predict the joint tensile wall thickness with the data of the ultrasonic testing experiment as input and the results of finite element simulation as output. The prediction effect of different neural network models was compared, and the results showed that the prediction and fitting effect of the SVM model was better, but overfitting occurred during the fitting process. Furthermore, particle swarm optimization is used to optimize the penalty parameter C and the kernel parameter g in the SVM model. Compared with the traditional SVM model, the PSO–SVM model is more suitable for the prediction of joint tensile wall thickness, which can better guide the production to solve this problem.

## 1. Introduction

**Figure 1:**
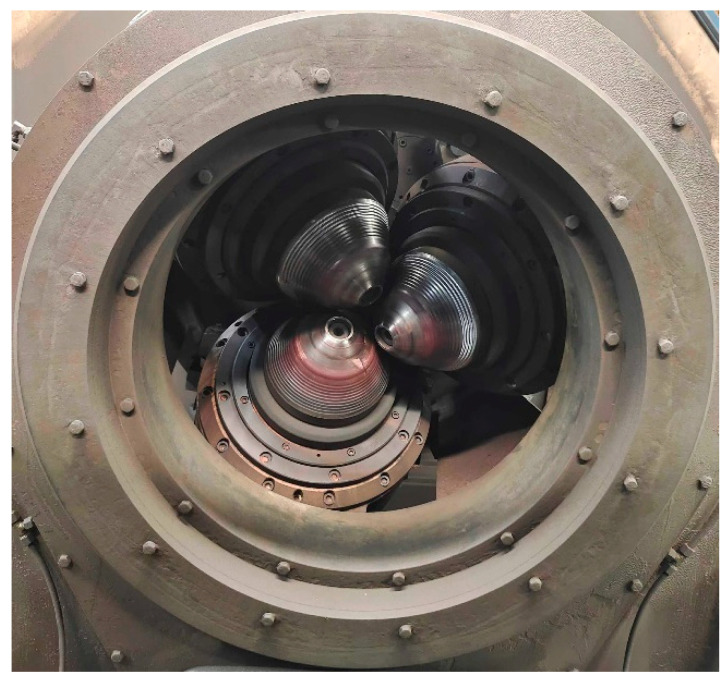
Three-roll rotary rolling.

TP2, as a kind of copper pipe, is composed of copper and a small amount of phosphorus, of which the copper content is usually more than 99.85%. TP2 copper pipes have excellent conductivity, thermal conductivity, corrosion resistance, and excellent processability and are widely used in fields such as air conditioning, refrigeration, aerospace, intelligent manufacturing, and computers [1,2,3,4]. Quality consistency is a bottleneck problem that restricts the application of TP2 copper pipes in complex working conditions. Three-roll rotary rolling is the second process in the production of TP2 copper pipes [5]. The metal billet is rolled continuously, mainly based on three horizontally aligned rolls to change its shape and size, as shown in Figure 1. During the rolling process, the metal billet is squeezed between three rollers in different directions, and due to the increasing pressure between the rollers, the metal billet is gradually squeezed into the desired shape and size. When the metal billet passes through the end of the three-high mill, its cross-sectional area is greatly reduced, while its length is significantly increased, resulting in large deformation, which easily causes uneven wall thickness defects and seriously affects the product quality of the subsequent process [6]. How to predict the wall thickness of copper tubes in subsequent processes is currently one of the key concerns in the field of copper tube casting and rolling processes.

There are many methods for non-destructive testing, and the most common ones can be divided into five types [7]: radiographic testing (RT); ultrasonic testing (UT); magnetic particle testing (MT); penetrant testing (PT); and eddy current testing (ECT). Among them, ultrasonic testing (UT) has the characteristics of strong penetration ability, accurate positioning, and high sensitivity. It can accurately locate defects inside and on the inner and outer walls of materials without damaging the pipes and is more suitable for the detection of precision pipes. Most scholars conduct defect detection research on materials or structures based on ultrasonic testing methods. Chen Shu et al. determined the working parameters of the water immersion-focused transverse wave method and water immersion-focused longitudinal wave direct injection method, conducted experiments on different types of defects, and obtained the advantages of water immersion-focused longitudinal wave direct injection method and water immersion-focused transverse wave method. The combination of the two methods can effectively improve the accuracy of detection [8]. Guo Zonghao et al. utilized the characteristics of the weld seam between the reactor pressure vessel connecting the pipe and the cylinder body and selected probes with different frequencies and angles for defect detection and quantification. The detection results met the requirements, and the automated ultrasonic testing of the inlet and outlet connecting pipe welds of the reactor pressure vessel could be achieved [9]. Fu Zongzhou conducted C-scan testing on the established standard test blocks to verify that the C-scan testing based on porosity standard test blocks could achieve monitoring and evaluation of the 2% porosity production line of carbon fiber composite laminates, meeting the requirements of engineering specifications [10]. Mortada et al. described ultrasonic testing technology and demonstrated the research progress made by air-coupled ultrasonic testing systems in the field of defect detection in composite material structure manufacturing [11]. Kumar et al. studied the propagation of ultrasonic waves on cracks through laboratory experiments and finite element models. For crack propagation beyond 20% crack depth, the transmission index is inversely proportional to the crack depth [12]. Tunukovic et al. developed an ultrasonic detection device provided by an industrial robotic arm, greatly improving detection speed and positioning accuracy [13]. Bilici used ultrasonic testing to evaluate the properties of composite materials and observed a linear relationship between grain size and physical and mechanical properties based on ultrasonic characteristics [14]. Tunukovic et al. compared the performance of object detection models, defect detection statistical methods, and traditional assignment threshold methods in carbon fiber defect detection. Intelligent machine learning improved amplitude threshold and statistical threshold techniques [15]. Liu Bo studied the water immersion ultrasonic C-scan detection method for open diffusion welded titanium alloy hollow support plates from the perspectives of theory and experimental verification. From the production of the reference block to the determination of the probe, water distance, gain, and gate, the rationality and reliability of the ultrasonic detection reference block and detection process were verified through comparative analysis [16]. Liu Ping distinguished the identification of residual height signals at the root of the weld seam, “mountain” wave signals, cover surface signals, and base metal reflection signals, which was beneficial for spectrum acquisition and correct judgment of defects [17]. Fadzil et al. used phased array ultrasonic testing technology and classic time correction gain method to detect good ultrasonic signals from the interface layer and back wall and compared them qualitatively and quantitatively with the proposed ultrasonic method, showing a high degree of consistency [18].

Numerous scholars have conducted extensive research on wall thickness changes. Zhang et al. have explored a new high-speed electrical discharge copper tube machining scheme, which simulates the deformation process of copper tubes during machining through finite element analysis. A prediction model for copper tube electrode wall thickness changes has been established, and the accuracy of the prediction has been verified by comparing it with experimental measurements [19]. Gao et al. conducted research on the reduction in outer wall thickness during the rotational bending process of eccentric pipe fittings. By establishing a finite element model, they analyzed the changes in axial and radial wall thickness and found the optimal eccentricity between the inner and outer centers of the pipe section [20]. Pavlov et al. studied the shape change in alloy steel pipes by using the method of computer simulation, and put forward the suggestion of selecting mandrel calibration in the longitudinal rolling process, thus improving the accuracy of pipes and reducing defects [21]. Shrivastava et al. explored the influence of different wall thickness changes on Al–Mg–Si alloy tubes by using numerical simulation and experimental comparison, and the results showed that when the tube wall thickness was the lowest, the deformation in the compression process was the largest [22]. Shemonaeva investigated the influence of circular pipe cross-section on the wall thickness distribution of the final part and found that the wall thickness quality of the finished part could be improved by generating a horizontal thickness difference [23].

The neural network prediction algorithm, with its powerful nonlinear mapping capabilities, self-learning, and adaptive characteristics, as well as its broad application scope, plays a crucial role in modern deep learning. As technology continues to develop and innovate, the application prospects of neural networks will expand even further. Liu et al., aiming to achieve accurate predictions of power grid failures, proposed a distribution network fault classification prediction model that combines a three-layer data-mining model (TLDM) with an improved backpropagation neural network (BPNN) enhanced by the adaptive moment estimation (Adam) algorithm and stochastic gradient descent [24]. Zhang et al. employed an improved support vector machine (SVM) method to construct a machine learning-based prediction model, enhancing the accuracy and efficiency of fatigue strength predictions for intercoolers [25]. Yin et al. used a radial basis function (RBF) neural network model to predict flame retardancy [26].

In summary, currently, ultrasound-based defect detection is mainly applied to simple regular structures such as flat plates, but for complex precision tube TP2 materials, ultrasound detection is still in the theoretical research stage and has not been applied in engineering practice. At present, static, offline, and destructive methods are mainly used in engineering for quality inspection of rotary rolled tube billets, which require inspection of cut tube billets. These methods have complex and cumbersome processes, low efficiency, and high randomness, and they cannot meet the real-time online inspection needs of the production process under the background of intelligent manufacturing requirements. In response to this issue, an intelligent online ultrasonic testing platform for wall thickness detection has been independently developed. Research on rolling wall thickness changes has been conducted, and the detection accuracy has been verified by comparing it with offline testing results. Finite element simulation has been used to obtain joint wall thickness data, and different neural networks have been used to predict joint wall thickness. The best prediction algorithm has been compared and optimized to obtain a more accurate prediction model.

## 2. Three-Roll Planetary Rotary Rolling Tube Billet Online Detection Experimental Platform

**Figure 2:**
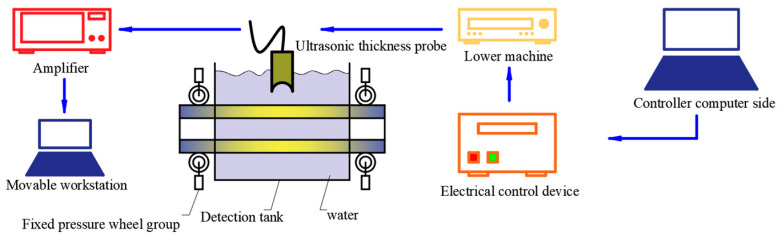
Ultrasonic experiment process after rolling.

To solve the problem of low efficiency and inaccurate detection of the wall thickness of three-roll planetary rotary rolling tube billets in existing technology, an intelligent online ultrasonic thickness measurement experimental platform has been independently developed. The detection process is shown in Figure 2. The electrical controller is controlled by the computer, and the electrical controller sends instructions to the lower computer. The lower computer then transmits the signal to the ultrasonic thickness measurement probe, generating ultrasonic waves.

### 2.1. Detection Device

**Figure 3:**
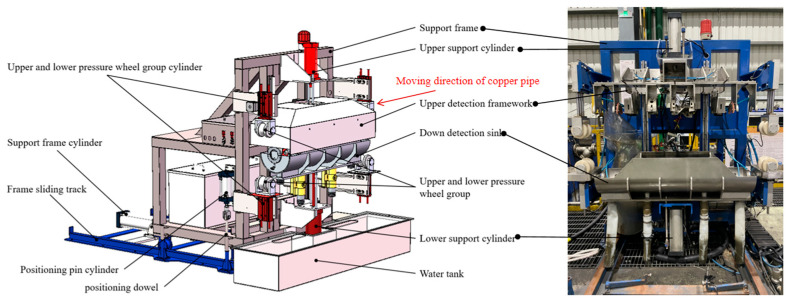
Wall thickness detection device.

In the production process of three-roll rotary rolling, the copper pipe is produced at a certain speed under the drive of the roll and the feeding trolley. In order to achieve the best production quality control, a set using ultrasonic sensors for measuring the pipe thickness in real time was developed (Figure 3). The measuring device is formed by a housing necessary to plunge the pipe into water, and the sensor is set to be placed around the pipe. The housing was installed next to the process line, which was composed of upper and lower containers mounted on a sliding support frame that can be set in and out of the process line. For measuring, the main frame is set in the line, and the containers are closed around the pipe. Two wheels at the entrance and the exit close, fixing the pipe. The closed housing is a sealed tank filled with water in a short time to the desired level. The measurement is performed with the UT probes, as described below. Once the test is finished, the water is drained and recycled for the next cycle, the tank opens, the wheels release the pipe, and the device can be removed from the line.

**Figure 4:**
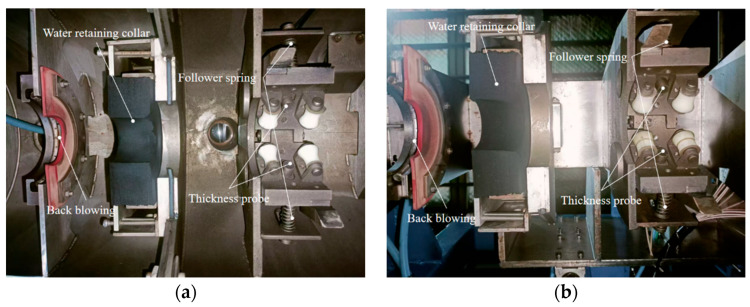
Probe position distribution: (**a**) Thickness probe set below; (**b**) Thickness measuring probe group ((**a**) is located in the Lower detection sink in Figure 3; (**b**) is located in the Upper detection framework in Figure 3).

Four probes are arranged at 90° around the axial direction to monitor the wall thickness of the rolled copper pipe in real time. In order to ensure the accuracy of the measuring probe, a follow-up device is installed at the position of the probe base. The follow-up device is connected to the probe base and the detection frame by a spring. When the copper tube is shaken or bent, the probe base is driven by the spring, ensuring that the probe and the follow-up device are relatively stationary along the radial direction so that the focus of the probe is always located on the axial center line of the rolled tube blank, improving the detection accuracy. The thickness measuring device is shown in Figure 4.

**Figure 5:**
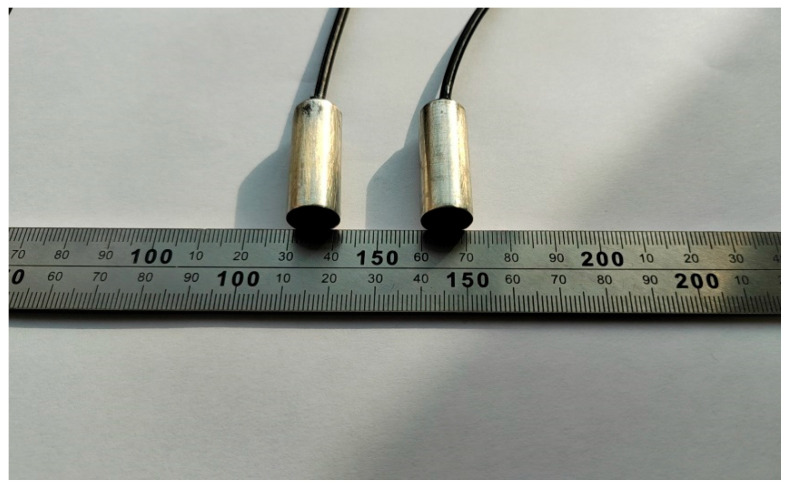
Thickness probe.

The ultrasonic thickness probe is made of a stainless steel shell and a composite circular wafer of a diameter of 10 mm, as shown in Figure 5. The probe parameters are listed in Table 1.

### 2.2. Data Analysis and Processing System

**Figure 6:**
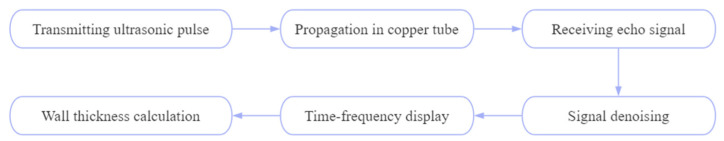
Data analyzing and processing system.

Ultrasonic thickness measurement is based on looking into the propagation through the workpiece and measuring the different acoustic paths caused by the boundary surfaces. This non-destructive method requires ultrasound pulse generation, transmission, reception, and signal processing, as shown in Figure 6. Signal processing mainly includes three aspects: signal noise reduction; time-frequency display; and wall thickness calculation.

#### 2.2.1. Wavelet Threshold Denoising

To obtain a high-quality signal, a denoising method is applied, using wavelet transform to decompose the initial signal. This method can be divided into the following four steps:

Step 1: Perform two-dimensional discrete wavelet transform (DWT) on the original image to obtain high-frequency and low-frequency components;

Step 2: Find the appropriate threshold. Calculate threshold values based on the relationship between signal frequency band and strength and process the decomposed high-frequency and low-frequency parts separately;

Step 3: Threshold the wavelet coefficients of high-frequency and low-frequency components according to the contraction criterion. Common methods include hard thresholding and soft thresholding;

Step 4: Perform wavelet inverse transform on the processed signal to obtain the denoised reconstructed image (IDWT):

(1) Wavelet Transform

After shifting the wavelet basis function φ(t) by β, perform inner product with the signal η(t) to be analyzed at different scales μ, such as [27]:

(1) $$DWT(\mu ,\beta )=\frac{1}{\sqrt{\mu }}\int_{-\infty }^{+\infty }\eta (t)\varphi (\frac{t-\beta }{\mu })dt$$

where μ is the scale factor; β is the translation factor; φ(t) is the conjugate function of the mother wavelet. Wavelet transform performs multi-scale analysis on the original signal η(t) by changing different scale factors μ, achieving filtering;

(2) Selection of Visu Universal Threshold

Visu universal threshold refers to using a pre-set fixed value as a threshold to threshold the coefficients after wavelet transform [28]:

(2) $$\lambda =\alpha \sqrt{2\ln(N)}$$

where α is the standard deviation of noise; *N* is the signal length;

(3) Threshold function

There are usually two forms of threshold functions: hard threshold and soft threshold. Among them, the hard threshold is [29]:

(3) $$\lambda (wt)=\left\{\begin{array}{l} \lambda (wt),\left|\lambda (wt)\right|>\lambda \\ 0,\left|\lambda (wt)\right|\leq \lambda \end{array}\right.$$

However, hard thresholding is prone to signal distortion. In the case of soft thresholding, the high-frequency coefficients are not completely retained when they are greater than the threshold. Instead, the coefficients are correspondingly reduced, making the signal smoother, better preserving the detailed information in the signal, and avoiding signal distortion:

(4) $$\lambda (wt)=\left\{\begin{array}{l} \mathrm{sgn}(\lambda (wt))(\left|\lambda (wt)-\lambda \right|),\left|\lambda (wt)\right|>\lambda \\ 0,\left|\lambda (wt)\right|\leq \lambda \end{array}\right.$$

(4) Inverse Wavelet Transform

Through inverse wavelet transform, the processed wavelet coefficients can be recombined into an approximation of the original signal, achieving noise reduction:

(5) $$x(\tau )\_new=\frac{1}{C_{\varphi }}\int_{0}^{+\infty }\int_{-\infty }^{+\infty }DWT_{\mu }^{\beta }x(\tau )\varphi_{\mu ,\beta }(t)d\mu d\beta$$

where $\varphi_{\mu ,\beta }(t)=\frac{1}{\sqrt{\mu }}\varphi (\frac{t-\beta }{\mu })$, and $C_{\varphi }$ is the constant of the wavelet function.

#### 2.2.2. Time-Frequency Display

The pulse–echo method for ultrasonic thickness measurement uses sharp pulses for excitation, and the obtained echo signal is an oscillating pulse signal. Its mathematical model can be expressed as [30]:

(6) $$h(t;\tau )=\beta e^{\alpha {\left(t-\tau \right)}^{2}}\sin(2\pi f_{c}(t-\tau )+\phi )$$

where β is the amplitude of the echo signal; α<0 is the broadband factor; τ is the delay parameter to be estimated; $f_{c}$ is the center frequency; ϕ is the phase of the ultrasound echo signal.

Wavelet transform is used to decompose a noisy signal, and threshold processing is applied to each layer of the decomposed coefficients to filter out noise-related wavelet coefficients. The denoised signal is then reconstructed. Formula (6) represents a cosine-modulated Gaussian envelope signal, which can be solved using the Fourier transform:


(7) $$\begin{array}{ll} H(\omega ) & =\int_{-\infty }^{+\infty }h(t)dt\\ & =\frac{\beta }{2i}\sqrt{-\frac{\alpha }{\pi }}\left[e^{\frac{{\left(2\pi f_{c}-\omega \right)}^{2}}{4\alpha }+i(\tau (2\pi f_{c}-\omega ))}-e^{\frac{{\left(2\pi f_{c}+\omega \right)}^{2}}{4\alpha }+i(\tau (2\pi f_{c}+\omega ))}\right] \end{array}$$

In engineering, only positive frequencies have physical significance; therefore, the final form of the Fourier transform of the reflection echo model is


(8) $$\begin{array}{l} \int_{-\infty }^{+\infty }y\cdot e^{i\omega t}dt\\ =\frac{\beta }{i}\sqrt{-\frac{\alpha }{\pi }}e^{\frac{{\left(2\pi f_{c}-\omega \right)}^{2}}{4\alpha }+i(\tau (2\pi f_{c}-\omega ))} \end{array}$$

The above equation shows that the amplitude spectrum of the ultrasonic echo model is still a Gaussian function, and the maximum value of the amplitude spectrum $\beta \sqrt{-\frac{\alpha }{\pi }}$ is obtained at the center frequency $\omega =2\pi f_{c}$.

#### 2.2.3. Thickness Measurement Principle

**Figure 7:**
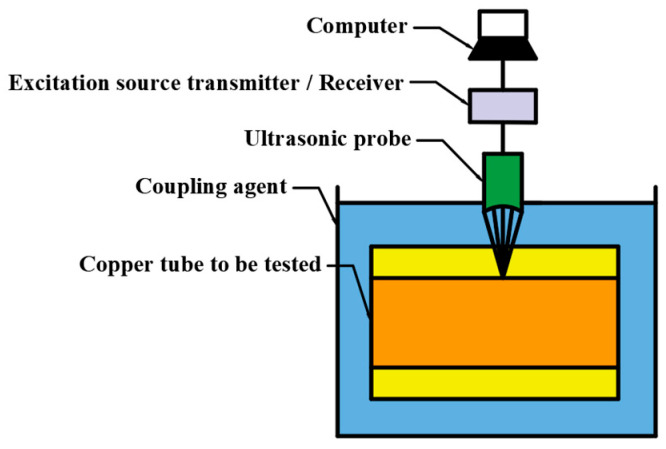
Ultrasonic thickness measurement by liquid immersion method.

According to the on-site inspection environment, the liquid immersion method is used for thickness measurement. The liquid immersion method is a non-contact thickness measurement method, in which a certain thickness of liquid medium exists between the ultrasonic transducer and the measured object, acting as an ultrasonic coupling agent, as shown in Figure 7.

The principle of liquid immersion thickness measurement is as follows: the plane wave emitted by the ultrasonic probe passes through the acoustic lens and the water immersion layer. If the velocity of the ultrasonic wave in the acoustic lens meets the velocity in the water immersion layer, the ultrasonic beam is focused. If focused thickness measurement is not used, then water immersion thickness measurement is the same as the common direct contact thickness measurement method; that is, the water layer only acts as a coupling agent. When calculating the thickness of the measured object, it is necessary to distinguish between the propagation time of ultrasound in the water layer and the measured object. The thickness calculation formula is as follows:

(9) $$D=\frac{1}{2}V_{L}\times (t_{1}-t_{2})$$

where D is the thickness of the measured object; $V_{L}$ is the propagation velocity of transverse waves in the measured object; $t_{1}$ is the round-trip time between the ultrasonic probe and the bottom surface of the object being measured; $t_{2}$ is the time it takes for the ultrasonic probe to travel back and forth between the upper and lower surfaces of the object being measured. The time-frequency signal is extracted, and the time values t_1_ and $t_{1}$ of the two echo peaks are calculated, $t_{2}$ being the round-trip time between the ultrasonic probe and the bottom surface of the object being measured and t_2_ being the time it takes for the ultrasonic probe to travel back and forth between the upper and lower surfaces of the object being measured. The time difference between the two echo peaks is used to calculate the wall thickness indirectly.

### 2.3. Experimental Verification

**Figure 8:**
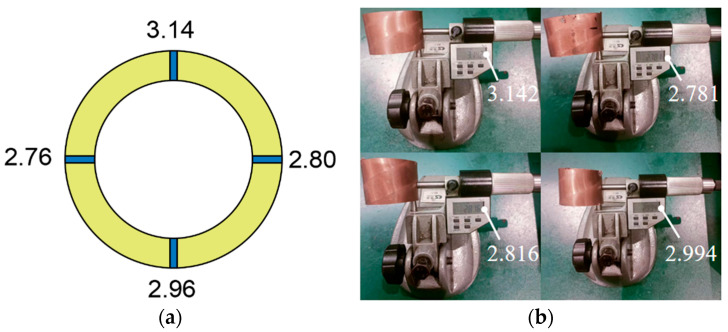
Comparison of wall thickness data: (**a**) Ultrasonic testing data; (**b**) Hand measurement data.

In order to verify the accuracy of the ultrasonic detection system, four different rolled sample tubes were measured with the new device after adjusting the gain and gate position and measuring four points on each sample, as shown in Figure 8. The samples were intercepted for offline measurement by direct contact with a spiral micrometer.

The accuracy of ultrasonic detection data is 0.01, and the precision of the spiral micrometer is 0.001. In order to reduce the errors caused by manual measurement, we measured three times at the same position, took the average value, calculated the relative errors of the two, and drew Table 2. Through statistics, we can see that the maximum relative difference between ultrasonic data and manual measurement data is 2.40%.

## 3. Establishment of Finite Element Model

### 3.1. Finite Element Model Building

**Figure 9:**
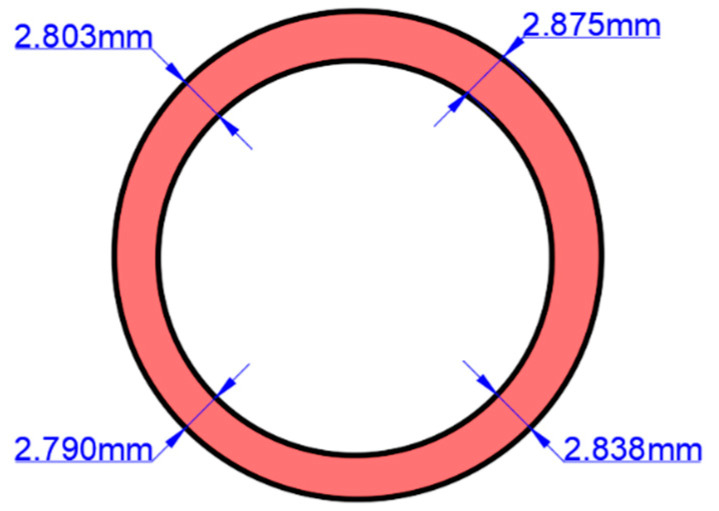
Establishment of initial tube blank.

For the FE model, the initial tube blank was set with a length of 50 mm and an outer diameter of 48.4 mm. A realistic thickness section of the pipe was defined according to the ultrasonic test results, using the average value of each test position, as shown in Figure 9.

#### 3.1.1. Model Construction of Deforming Body and Mold

**Figure 10:**
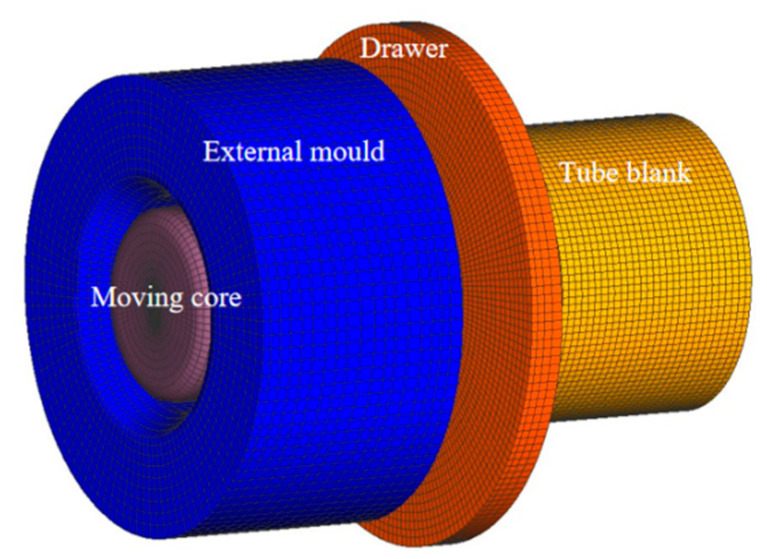
Initial finite element model of pipe.

The finite element model shown in Figure 10 consists of four parts: the floating core head; the outer mold; the drawing tool; and the tube blank. The tube blank gradually contacts the floating core head and the outer mold under the action of the drawing tool, entering the deformation area under their combined effect. When the tube blank enters the outer mold area, the outer diameter of the tube blank tightly contacts the mold due to the smaller inner diameter of the mold, resulting in radial compression for diameter reduction. Simultaneously, the inner wall gradually contacts the core head, expanding outward and reducing wall thickness. The core head adjusts its position based on the deformation of the tube blank to maintain uniform support internally. An initial tube blank size of *Φ* 48.4 × 2.8 mm is reduced to *Φ* 38 × 2.2 mm after the first drawing and, finally, to *Φ* 30.03 × 1.605 mm after the second drawing. Since the deformation is axial and axisymmetric, the model is divided into 33,000 hexahedral mesh elements with a size of 1.17 mm. The drawing mold material is carbide YG8, with the outer mold defined as the deformable body, the core head as a rigid body, and spring-driven to simulate its axial position adaptability.

#### 3.1.2. Setting Pipe Parameters

The mass density of the TP2 copper tube is ρ = 8940 kg/m^3^; the thermal conductivity is λ = 390 W/(m·k); the specific heat capacity is c = 400 J/(kg·k); the elastic modulus is E = 1.170 × 10^5^ MPa, and the Poisson’s ratio is ν = 0.33. The drawing speeds of the two passes were 48 and 72 m/min, respectively, and the friction coefficient was 0.05. The coefficient of thermal expansion varies with temperature, and the specific values are shown in Table 3.

#### 3.1.3. Pipe Performance Parameter

To determine the material properties, a uniaxial stress–strain test was performed with an electronic universal testing machine at a deformation speed of 6 mm/min and repeated three times, yielding a load-displacement curve.

After exceeding the elastic limit due to the significant size change (reduction in diameter), the true stress formula $\sigma_{T}=P/A$ is used instead of the engineering stress formula $\sigma_{e}=P/A_{0}$ to more accurately measure the material response in the plastic flow range. The true strain and true stress formulas are as follows:

(10) $$\varepsilon_{T}=\ln(\frac{I_{0}+\Delta I}{I_{0}})=\ln(1+\varepsilon_{e})$$

(11) $$\sigma_{T}=\sigma_{e}(1+\varepsilon_{e})$$

where $I_{0}$ is the initial gauge length of the sample; ΔI is the elongation of the gauge; $\sigma_{e},\varepsilon_{e}$ represent engineering stress and engineering strain; $\sigma_{T},\varepsilon_{T}$ represent true stress and true strain.

**Figure 11:**
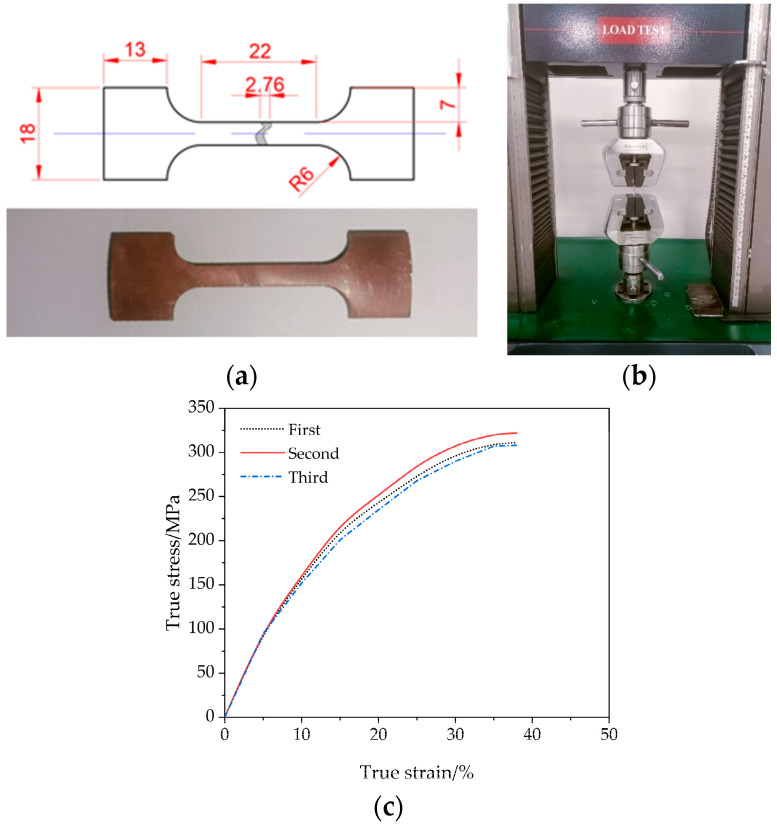
Tensile test: (**a**) Tensile specimen of TP2 copper; (**b**) Electronic universal testing machine; (**c**) Real stress–strain curve measured for 3 specimens.

The true stress–strain curve of the initial tube blank is shown in Figure 11 for three different specimens. The yield strength and tensile strength (average) values measured were 301 MPa and 319 MPa, respectively.

After obtaining the true stress–plastic strain curve, the Holloman hardening equation is applied to uniformly fit the true stress–strain curve to be the constitutive model of the following simulation. The Holloman constitutive equation is as follows [31]:

(12) $$\sigma_{T}=K\varepsilon_{T}^{n}$$

where K is the strength coefficient, and n is the strain hardening index.

By fitting Holloman’s constitutive equation, the fitting degree is 98%, and the coefficients of K and n are 81.7 and 0.36, respectively.

According to the research by Liu et al. [32], the actual drawing temperature ranges from 65.9 °C to 108.5 °C, and the effect of temperature on the model is minimal. Additionally, as shown in Chen’s study [33], the yield strength of the tube does not vary significantly between 20 °C and 100 °C. Therefore, data collected at room temperature can be used for simulation.

### 3.2. Comparison of Finite Element Results

**Figure 12:**
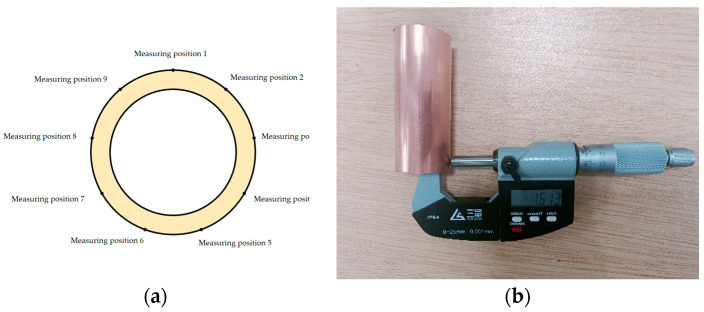
Wall thickness measurement: (**a**) Finite element model wall thickness data; (**b**) Spiral micrometer measurement data.

After the simulation, nine tracking points are established along the same cross-section to extract wall thickness data via post-processing of the finite element model; the drawn copper tube segment is measured for thickness using a spiral micrometer at nine points along the same section, as shown in Figure 12 and Table 4.

As can be seen from Table 5, the maximum wall thickness deviation between the finite element simulation data and the actual production manual data is 0.045 mm.

## 4. Neural Network Prediction

The wall thickness of four locations detected by ultrasound was used as the input of the neural networks (NNs). The corresponding wall thickness provided by the FE simulation (using the same input thickness data) was used as the output of the NNs. A total of 400 sets of wall thickness data were measured using the new equipment in the process line, as shown in Table 5, and the corresponding 400 evaluations performed by the FE model were calculated. According to the prediction method proposed by Liu, Zhang, Yin et al. [25,26,27], the BP neural network, SVM neural network, RF neural network, and RBF neural network are used for prediction. Three hundred sets of data are randomly selected as the training sets of this model, 60 sets of data as the test sets, and the remaining 40 sets as the verification sets. Different types of neural networks and machine learning models are implemented using the Scikit-Learn learning library in the Python 3.10 language.

### 4.1. BP Neural Network

**Figure 13:**
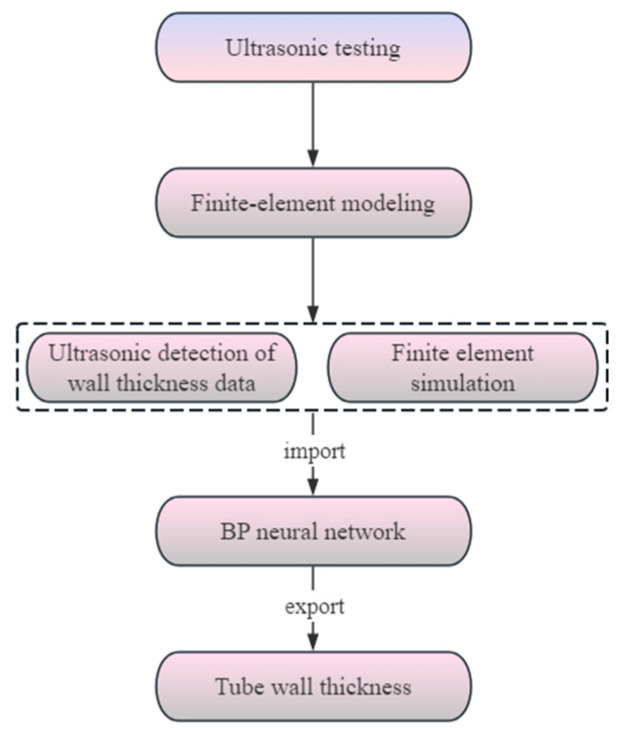
Predictive implementation framework.

The BP neural network (Back Propagation Neural Network) mainly consists of three parts: input layer; hidden layer; and output layer [30,34], and the main factors affecting the wall thickness of copper pipe after joint drawing are uneven radial wall thickness of copper pipe after rolling. The wall thickness of the input layer is measured at four different positions by an ultrasonic testing platform, and the wall thickness of an output layer is the wall thickness of copper pipe after joint drawing. The BP neural network prediction framework is shown in Figure 13.

### 4.2. SVM Model

SVM (Support Vector Machine) is suitable for small sample learning algorithms, which can project samples from low-dimensional space to high-dimensional space in a nonlinear manner, thereby transforming linear optimization problems into convex programming problems and obtaining the optimal solution of the original problem [35,36]. At the same time, kernel functions are used to convert inner product operations in high-dimensional space into kernel function operations in the original space.

If the training samples have nonlinear relationships, each set of data in the training samples can be transformed into a high-dimensional space, and linear regression can be performed on the data in the high-dimensional space, thereby transforming the nonlinear fitting problem of the original samples into a linear regression problem of the samples in the high-dimensional space. The obtained fitting function is [37]:

(13) y=ω⋅ψ(x)+b

where x is the input vector; y is the output vector; ω is the weight vector; b is the fitting deviation; ψ(x) is a nonlinear mapping. By training ω and b multiple times in Equation (13), the minimum values of Equations (14) and (15) can be obtained:

(14) $$Q(f)=c\sum_{i=1}^{n}\varsigma (f(x_{i})-y_{i})+\frac{1}{2}\omega^{2}$$

(15) $$\varsigma (f(x_{i})-y_{i})=\frac{1}{n}\left\{\begin{array}{l} \left|f(x_{i})-y_{i}\right|-\vartheta \left|f(x_{i})-y_{i}\right|\geq \vartheta \\ \left|f(x_{i})-y_{i}\right|<\vartheta \end{array}\right.$$

where $c\sum_{i=1}^{n}\varsigma (f(x_{i})-y_{i})$ is the empirical error of the optimization problem; $\frac{1}{2}\omega^{2}$ is the normalization parameter of the optimization problem; ς(⋅) is the cost parameter of the optimization problem; c is the penalty factor for the optimization problem; ϑ is the loss function parameter for the optimization problem.

To achieve the transformation from nonlinear to linear problems and transform the learning process of support vector machines into computable convex optimization problems, the following equation is used:

(16) min12w2+C∑i=1l(ξi+ξi)*;s.t.yi−wϕ(xi)−b≤ε+ξi;−yi−wϕ(xi)+b≤ε+ξi,i=1,2,…,l;ξi≥0,ξi≥*0.

where C is the penalty coefficient. To simplify the prediction process, it is transformed into a dual problem:

(17) max∑i=1nyi(ai−ai)*−ϑ∑i=1n(ai+ai)*−12∑i=1n∑j=1n(ai−ai)*(aj−aj)*K(xi,xj)s.t∑i=1nai−ai*≤ai,ai≤*c

where $a_{i}$, $a_{i}^{*}$ are the Lagrange multipliers in quadratic programming. According to Equations (13)–(17), the final regression function can be obtained as

(18) f(x)=∑i=1l(ai−ai)*K(xi,xj)+b

where ϕ(x) is a nonlinear mapping function; w is the direction vector; b is the intercept of the regression function; ε is the insensitivity coefficient; $K(x_{i},x_{j})=\phi (x_{i})\phi (x_{j})$ is the kernel function that satisfies the SVM condition; $x_{i}$ and $x_{j}$ are sample vectors; $y_{i}$ is the category of the training sample; $\xi_{i}$, $\xi_{i}^{*}$ is a relaxation variable.

On the basis of Equation (18), the radial basis function kernel function is used to simplify the solution, and the final kernel function is as follows:

(19) $$K(x_{i},x_{j})=\exp(-g{\left\|x_{i}-x_{j}\right\|}^{2}),g>0$$

where g is the nuclear parameter.

### 4.3. RF Neural Network

**Figure 14:**
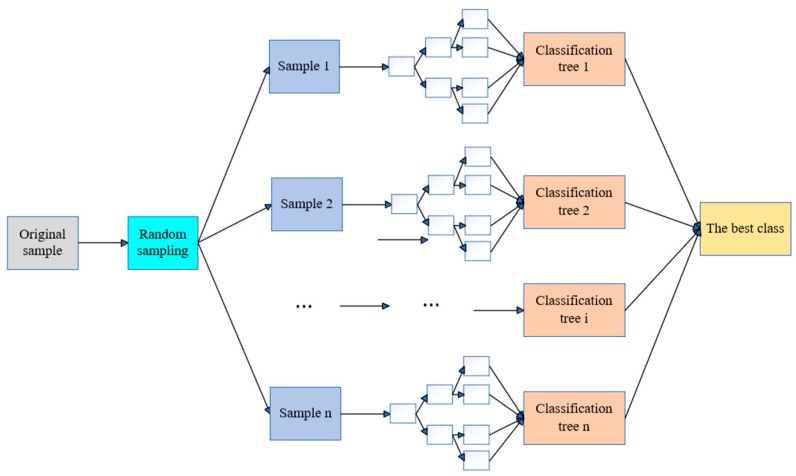
RF neural network topology.

Random forest (RF) is a machine learning algorithm based on decision trees developed by Breiman [38], which can be used for classification, regression, and multidimensional data processing. It not only balances the errors of unevenly distributed data in the sample but also has a good tolerance for outliers and noise [39], as shown in Figure 14. From Figure 13, it can be seen that the RF model is composed of multiple classification trees 1, 2, …, n, and each decision tree works together to mine indicators that have a significant correlation with the model’s prediction accuracy.

### 4.4. RBF Neural Network

Based on the RBF (Radial Basis Function) neural network prediction of wall thickness uniformity, the quantitative factors affecting the wall thickness of the copper tube after joint drawing in this paper are the wall thicknesses at different positions of the rolled copper tube. A functional relationship between the factors affecting the wall thickness of the copper tube after joint drawing and the wall thickness is established:

(20) $$f_{s}=f(x1,x2,x3,x4)$$

where *x*1, *x*2, *x*3, *x*4 refer to the wall thicknesses at different positions of the rolled copper tube, and $f_{s}$ is the uniformity of the wall thickness of the drawn copper tube.

**Figure 15:**
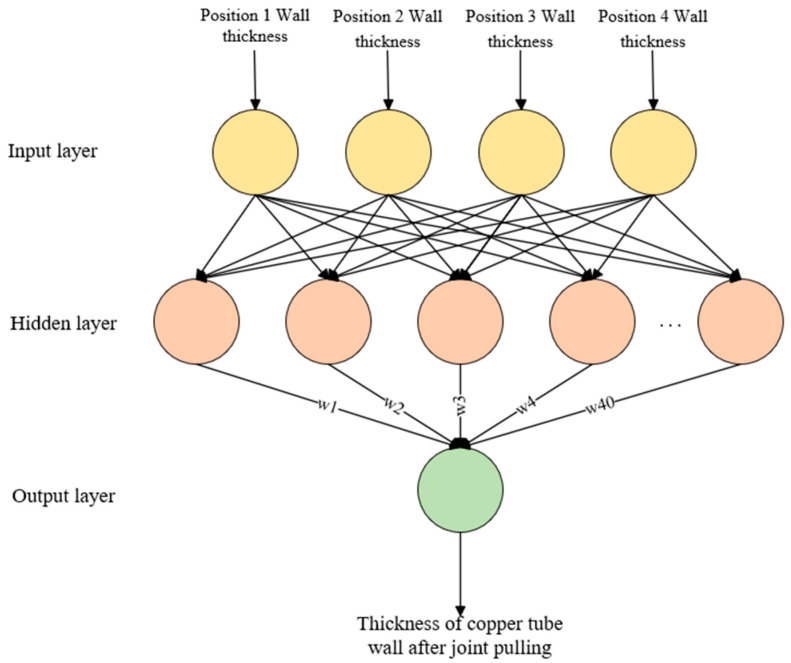
RBF neural network topology.

Therefore, this article takes the wall thickness of copper pipes at four different positions as the input layer of the neural network, one hidden layer, and the output layer as the wall thicknesses of the connected copper pipes. The topology structure of the RBF neural network is shown in Figure 15.

### 4.5. Evaluation Methods

The prediction of joint tensile thickness using the BP neural network, SVM neural network, RF neural network, and RBF neural network, respectively, belongs to the problem of regression prediction. After the predicted value is output, it needs to be compared with the real value to evaluate the fitting effect of this model. There are several commonly used methods to evaluate model accuracy: mean square error (*MSE*); root mean square error (*RMSE*); mean absolute error (*MAE*); determination coefficient (*R*^2^) [40,41]. For each evaluation index containing n samples, the formula is as follows:

- Mean Squared Error (*MSE*):

(21) $$MSE=\frac{1}{n}{\sum_{i=1}^{n}\left(y-y_{i}\right)}^{2}$$

- Root Mean Squared Error (*RMSE*):

(22) $$RMSE=\sqrt{\frac{1}{n}{\sum_{i=1}^{n}\left(y-y_{i}\right)}^{2}}$$

- Mean Absolute Error (*MAE*):

(23) $$MAE=\frac{1}{n}\sum_{i=1}^{n}\left|y-y_{i}\right|$$

- Determination coefficient (*R²*):

(24) $$R^{2}=1-\frac{{\sum \left(y_{i}-y\right)}^{2}}{\sum {\left(y_{i}-\bar{y}\right)}^{2}}$$

where y is the predicted value; $y_{i}$ is the actual value, and $\bar{y}$ is the mean of the predicted values. In this study, *MAE*, *MSE*, *RMSE*, and *R*^2^ are selected as the final evaluation metrics for the model. The smaller the values of *MAE*, *MSE*, and *RMSE*, and the closer *R*^2^ is to 1, the smaller the model’s error and the better the predictive performance.

### 4.6. Comparative Analysis of Neural Networks

**Figure 16:**
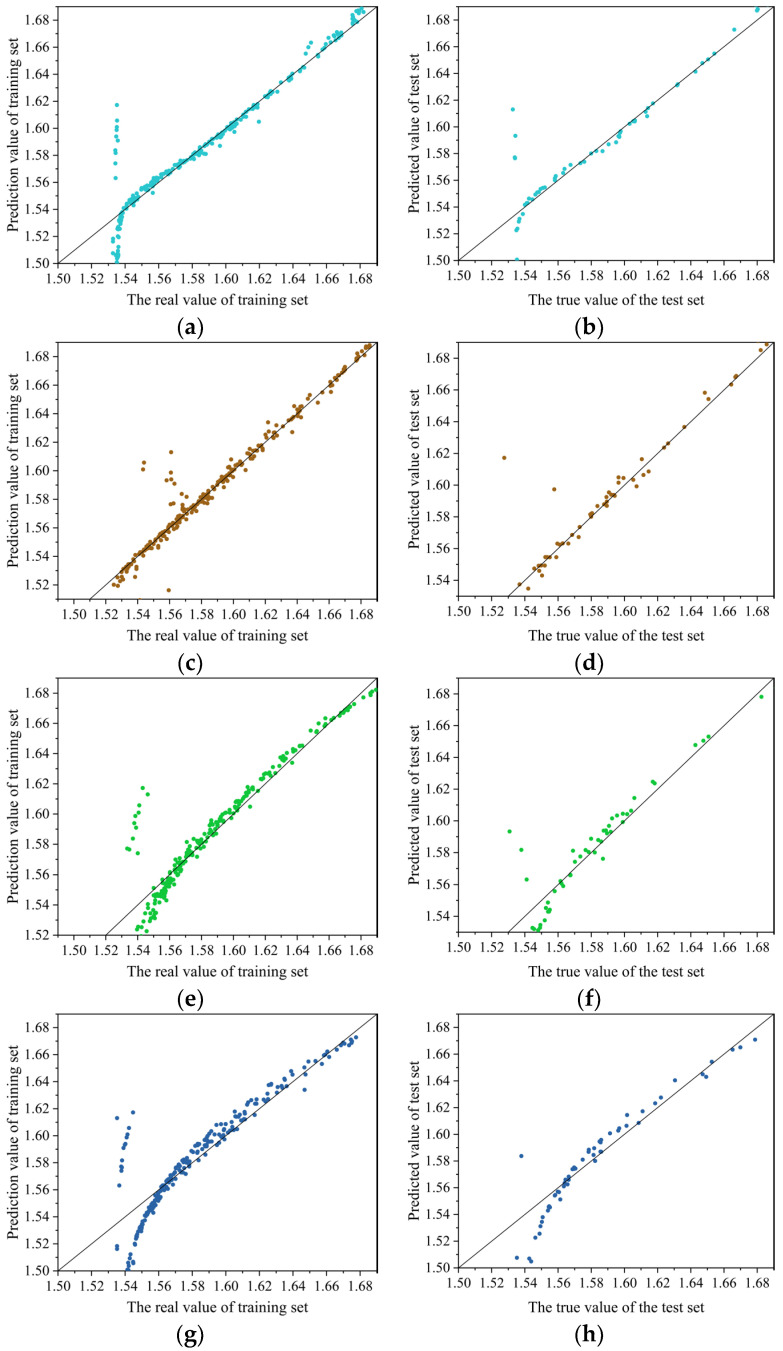
Different neural network verification results: (**a**) BP neural network training set; (**b**) BP neural network test set; (**c**) SVM neural network training set; (**d**) SVM neural network test set; (**e**) RF neural network training set; (**f**) RF neural network test set; (**g**) RBF neural network training set; (**h**) RBF neural network test set.

Figure 16 shows the predictive performance of the model on the training and testing sets: *SVM* > *BP* > *RF* > *RBF*. Table 6 presents the evaluation parameters *MAE*, *MSE*, *RMSE*, and *R*^2^ values for different models. In terms of prediction accuracy, the *SVM* model has high prediction accuracy (test set: *MAE* = 5.1 × 10^−3^, *MSE* = 2.8 × 10^−3^, *RMSE* = 1.21 × 10^−2^, *R*^2^ = 9.23 × 10^−1^). SVM transforms the prediction problem into a convex optimization problem, ensuring that the found solution is the global optimal solution, while the application of kernel functions maps the sample space to higher dimensions, improving the model’s generalization ability. The prediction accuracy of the BP model (test set: *MAE* = 6.7 × 10^−3^, *MSE* = 3.5 × 10^−3^, *RMSE* = 1.97 × 10^−2^, *R*^2^ = 8.81 × 10^−1^) is slightly lower than that of the SVM model. This may be due to the fact that the BP neural network relies on an error backpropagation algorithm to adjust weights and biases to predict errors at the minimum value. However, this gradient descent-based prediction method is prone to getting stuck in local minima, resulting in the model being unable to reach the global optimal solution. The RF model (test set: *MAE* = 8.9 × 10^−3^; *MSE* = 4.6 × 10^−3^; *RMSE* = 2.09 × 10^−2^; *R*^2^ = 7.65 × 10^−1^) demonstrated stable predictive ability, but there may be some errors in the low wall thickness region. The prediction accuracy of the RBF model (test set: *MAE* = 1.1 × 10^−2^; *MSE* = 5.7 × 10^−3^; *RMSE* = 2.32 × 10^−2^; *R*^2^ = 7.21 × 10^−1^) is the lowest. This may be because the variance parameter of the Gaussian kernel function used in the RBF model controls the radial action range of the function [42]. Setting the variance too large or too small can affect the network’s adaptability to data, resulting in inferior performance in predicting the thickness of the connected wall compared to the other three models. However, in the four models, when the wall thickness is less than 1.55 mm, the prediction value is small, but when the wall thickness is greater than 1.55 mm, the four models have better prediction accuracy. It can be seen that the SVM neural network, BP neural network, RF neural network, and RBF neural network have higher accuracy in predicting large wall thickness, but when predicting small wall thickness, the prediction value of the SVM neural network is higher than that of BP neural network. SVM neural network has more stable prediction accuracy.

### 4.7. Comparison and Verification of Prediction Accuracy

**Figure 17:**
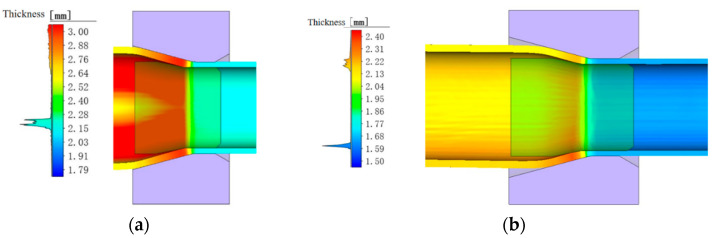
Finite element simulation of wall thickness: (**a**) the first pull wall thickness; (**b**) the second pull wall thickness.

Through a comprehensive comparison of evaluation metrics, the SVM neural network provided the highest prediction accuracy for this model. As an example and to verify the better prediction accuracy of the SVM neural network, the first set of data (group 1 in Table 5) is used to present the simulation results (Figure 17) and the comparison of the prediction results according to the different NNs (Table 7).

As can be seen from Figure 17a, when the tube billet enters the sizing section, the wall thickness begins to decrease, gradually decreasing from 2.8 mm to about 2.2 mm. In the double draw, the wall thickness is reduced from 2.2 mm to 1.6 mm. After the simulation, tracking points were established along any cross-sections, and the average wall thickness of the cross-section was calculated to be 1.611 mm.

Using the wall thickness from the finite element model as input, different neural networks were used for prediction (Table 7). As shown in Table 7, the wall thickness predicted by the SVM neural network for this copper tube is 1.606 mm, while the predictions from BP, RF, and RBF neural networks are 1.601 mm, 1.598 mm, and 1.587 mm, respectively, indicating that SVM provides higher predictive accuracy for practical production.

## 5. Particle Swarm Optimization Support Vector Machine Model

**Figure 18:**
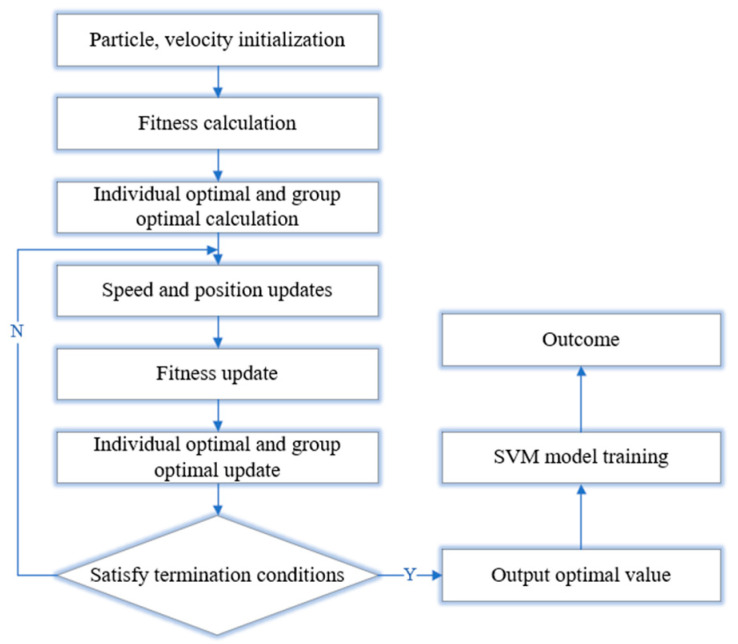
PSO–SVM prediction flow chart.

The penalty parameter *C* and kernel parameter *g* of the SVM algorithm directly affect the model’s prediction accuracy. To improve prediction accuracy, Particle Swarm Optimization (PSO) is first used to optimize *C* and *g*, and then the SVM algorithm is applied for training and prediction on the relevant data. The prediction flow of this study is shown in Figure 18.

### 5.1. PSO Optimization Algorithm

The most common optimization methods for support vector machine algorithms include genetic algorithm (GA) [43], least squares algorithm (LS) [44], particle swarm optimization algorithm (PSO) [45], etc. PSO (Particle Swarm Optimization) algorithm is an optimization method based on particle analysis. In the process of application, particles can determine their motion patterns based on their own group characteristics [46], thereby achieving the goal of optimization. By simulating the social behavior of bird or fish flocks, global search is achieved, avoiding the randomness of adjusting control parameters and reducing the probability of falling into local optimal solutions [47]. Particle iterative updates can be expressed as

(25) vi=LBωV⋅vi+LBc1rand(1)⋅(PbestLB−xi)LB+c2rand(2)⋅(GbestLB−xi)LBxi=LBxi+LBωPviLB

where $v_{i}^{LB}$ is the iteration speed of the LB-th generation; $x_{i}^{LB}$ is the particle position of the LB-th generation; $Pbest^{LB}$ is the individual optimal position of the LB-th generation, and $Gbest^{LB}$ is the global optimal position of the LB-th generation. LB=1,2…, i=1,2…, ωV is the inertia weight factor for velocity; ωP is the inertia weight factors for position; $c_{1},c_{2}$ are acceleration constants, and rand(1),rand(2) are random functions.

The PSO algorithm is implemented in Python using the pyswarm library. In order to optimize the accuracy and modeling efficiency of the SVM model, this experiment uses the PSO algorithm to optimize the penalty factor C and kernel parameter g. The PSO algorithm parameters are set as follows: the number of particles is 10; the maximum number of iterations of the particle swarm is 100; the acceleration factors are $c_{1}$ = 1.5 and $c_{2}$ = 1.7. The inertia weight factors are ωV and ωP are 1, respectively. The search range for both penalty parameter C and kernel parameter g is (0.1–100).

### 5.2. SVM Kernel Function Selection

**Figure 19:**
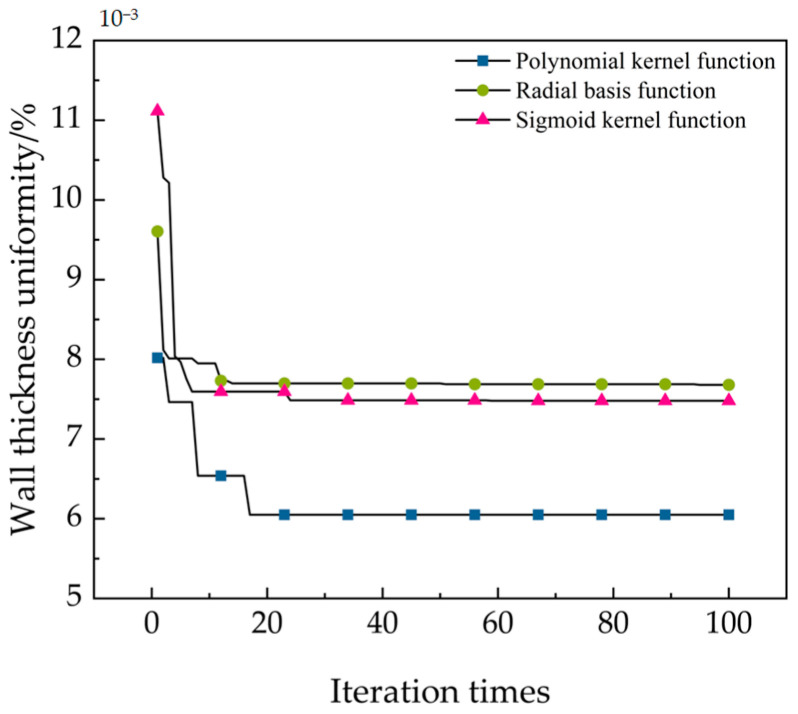
Variation curve of fitness.

In this study, the PSO algorithm was used to optimize the penalty parameter *C* and kernel function *g*. Three types of kernel functions, polynomial kernel function, radial basis kernel function, and sigmoid kernel function, were used for learning [48,49]. The best fitness of the predicted samples was used as the validation criterion to select the optimal kernel function type. The optimal fitness changes in the three types of kernel functions are shown in Figure 19. It can be seen that when the number of iterations reaches about 15, the three kernel functions gradually converge. From the entire result, it can be seen that using a polynomial as the kernel function has the best optimization effect. Therefore, a polynomial is chosen as the kernel function for the SVM algorithm in this study.

### 5.3. Verification of Prediction Results

**Figure 20:**
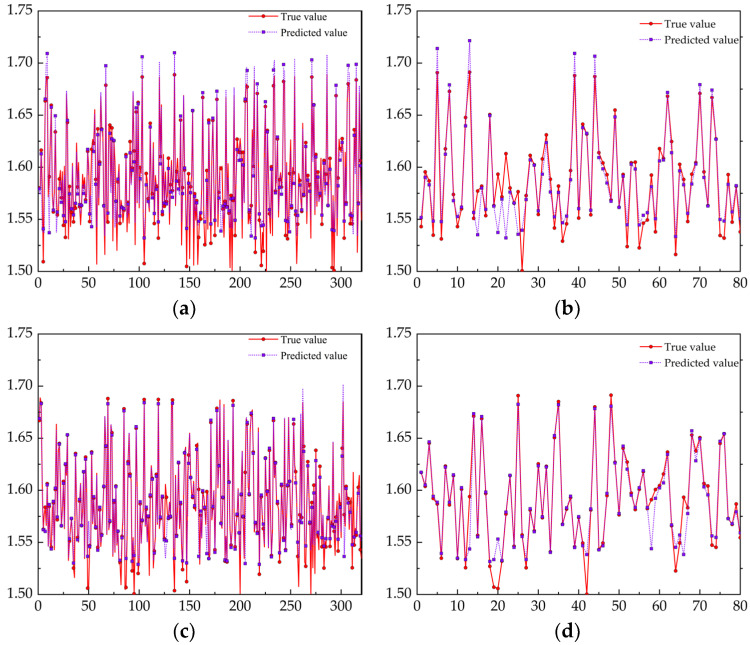
The prediction results of the two models: (**a**) SVM training set; (**b**) SVM test set; (**c**) PSO–SVM training set; (d) PSO–SVM test set.

The SVM model and PSO–SVM model were used to predict the joint tensile wall thickness. Three hundred and twenty sets of data were randomly selected as the training set, and 80 sets of data as the test sets. The prediction results are shown in Figure 20.

The predicted values of the two models are basically consistent with the real situation. The SVM neural network has a small prediction effect when the wall thickness is small, while the real values and predicted values of the PSO–SVM training set and test set have a better fitting degree. Moreover, the prediction results of the two models only have errors in the detail fluctuations, so it is considered that both models have the ability to predict the wall thickness. The evaluation parameters are shown in Table 8. After using the PSO algorithm to improve the traditional SVM model, the *RMSE* and *MAE* of the PSO–SVM model in the training set are, respectively, 1.08 × 10^−3^ and 2.0 × 10^−3^, and the *R^2^* of PSO–SVM is 9.64 × 10^−1^. Compared with the traditional SVM model, the improved PSO–BPNN model has better evaluation results. However, for the test set, *RMSE* and *MAE* of the PSO–SVM model were 1.45 × 10^−2^ and 3.4 × 10^−3^, respectively, and *R^2^* of the test set was 9.49 × 10^−1^, which had a good consistency with *R*^2^ of the training set sample (9.64 × 10^−1^). This indicates that compared with the traditional SVM model, the improved PSO–SVM model has a more stable prediction effect on wall thickness prediction. In the traditional model, the values of penalty parameter *C* and kernel function *g* are randomly selected, but the PSO–SVM model selects the optimal values of penalty parameter *C* and kernel function *g* through the PSO optimization algorithm. Therefore, the prediction effect of PSO–SVM is better.

### 5.4. Prediction Result Verification

**Figure 21:**
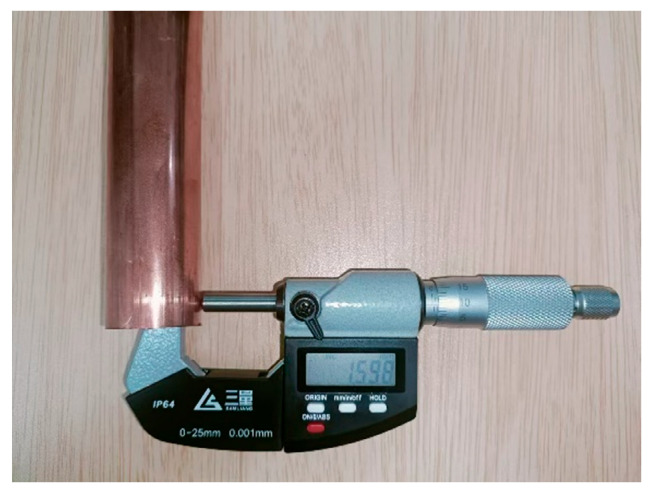
Production measurement.

Through the prediction of different neural networks, it is concluded that the PSO–SVM neural network is more suitable for joint drawing wall thickness prediction. In order to verify that the prediction results of the PSO–SVM neural network can better guide production, ultrasonic detection is used to detect copper pipes in production. After the detection, the copper pipes in the corresponding joint drawing process are intercepted. The spiral micrometer was used to measure the wall thickness of nine radial positions and calculate the average value, as shown in Figure 21. After the measurement, the data of four different positions detected by ultrasound were, respectively, input into five different neural networks, and the predicted values of different neural networks were obtained, as shown in Table 9.

As shown in Table 9, compared with the actual production data, the prediction results are all small, but the prediction deviation of PSO–SVM is at least 0.003 mm, indicating that the prediction effect of PSO–SVM is highly accurate and can guide production more effectively.

## 6. Conclusions

(1) The established online ultrasonic thickness test bench was used to detect the rolled copper pipe, and the maximum relative error between the ultrasonic data and the offline manual data was 2.40%, which had good accuracy and could meet the actual engineering requirements. The experimental equipment effectively solves the problems of low accuracy, low efficiency, and poor timeliness in the traditional method;

(2) The BP neural network model, SVM model, RF neural network model, and RBF neural network model were used for prediction, and the *R*^2^ of the training set were 8.86 × 10^−1^, 9.65 × 10^−1^, 8.71 × 10^−1^ and 7.67 × 10^−1^, respectively. The results showed that the above neural network models could effectively predict the wall thickness of the connecting copper pipe. But the SVM model has higher prediction accuracy;

(3) The *RMSE* of the test set of the PSO–SVM model decreased from 1.82 × 10^−2^ to 1.45 × 10^−2^; *MAE* decreased from 5.9 × 10^−3^ to 3.4 × 10^−3^, and *R*^2^ increased from 9.12 × 10^−1^ to 9.49 × 10^−1^. It can be seen that the PSO–SVM model has better stability than the traditional SVM model in predicting joint tensile wall thickness;

(4) The construction of an ultrasonic online thickness test bench and the prediction of PSO–SVM for joint tensile wall thickness data can better monitor the change in wall thickness on site. When the wall thickness deviation of the rolling mill is large, which has a serious impact on the joint tensile wall thickness, the roll can be adjusted in time to improve the copper pipe quality correlation coefficient.

## Figures and Tables

**Table 1:** Probe working information.

Item	Argument
Probe working mode	Single crystal
Damping	50 Ω
Frequency	10 MHz
Probe type	Water immersion line focusing probe
Probe Angle	90°
Channel gain	50 dB

**Table 2:** Sample tube data comparison.

Sample Tube	Detection Position	Ultrasonic Data	Hand Measured Data	Relative Difference
Sample tube 1	Slot 1	3.14	3.138	0.06%
	Slot 2	2.80	2.836	1.27%
	Slot 3	2.96	2.965	0.17%
	Slot 4	2.76	2.767	0.25%
Sample tube 2	Slot 1	2.98	3.049	2.26%
	Slot 2	3.14	3.118	0.71%
	Slot 3	2.92	2.944	0.82%
	Slot 4	3.14	3.112	0.90%
Sample tube 3	Slot 1	3.04	3.034	0.20%
	Slot 2	3.14	3.085	1.78%
	Slot 3	2.98	2.996	0.53%
	Slot 4	3.04	3.032	0.26%
Sample tube 4	Slot 1	3.14	3.127	0.42%
	Slot 2	3.03	2.959	2.40%
	Slot 3	2.98	2.939	1.40%
	Slot 4	2.82	2.828	0.28%

**Table 3:** Effect of temperature on thermal expansion coefficient.

Temperature/°C	0	100	200	300
Thermal Expansion Coefficient/μm/(m·k)	17.0	17.0	17.3	17.7

**Table 4:** Comparison of wall thickness data (mm).

Measurement Location	Finite Element Model Wall Thickness Data	Spiral Micrometer Measurement Data	Wall Thickness Deviation
1	1.602	1.621	0.019
2	1.625	1.605	0.02
3	1.614	1.624	0.01
4	1.606	1.651	0.045
5	1.609	1.644	0.035
6	1.608	1.617	0.009
7	1.625	1.621	0.004
8	1.600	1.641	0.041
9	1.612	1.621	0.006

**Table 5:** Wall thickness data statistics (mm).

Number of Groups	Wall Thickness at Position 1	Wall Thickness at Position 2	Wall Thickness at Position 3	Wall Thickness at Position 4	Joint Wall Thickness
1	2.838	2.875	2.803	2.799	1.611
2	2.856	2.884	2.838	2.819	1.623
3	2.867	2.885	2.863	2.835	1.631
…	…	…	…	…	…
399	2.872	2.781	2.799	2.815	1.625
400	2.835	2.798	2.819	2.845	1.629

**Table 6:** Wall thickness data statistics (mm).

Model	Training Set				Test Set			
	MAE	MSE	RMSE	R^2^	MAE	MSE	RMSE	R^2^
BP	4.7 × 10^−3^	4.3 × 10^−4^	1.29 × 10^−2^	8.86 × 10^−1^	6.7 × 10^−3^	3.5 × 10^−3^	1.97 × 10^−2^	8.81 × 10^−1^
SVM	4.2 × 10^−3^	8.7 × 10^−6^	1.12 × 10^−2^	9.15 × 10^−1^	5.1 × 10^−3^	2.8 × 10^−3^	1.21 × 10^−2^	9.23 × 10^−1^
RF	5.7 × 10^−3^	6.1 × 10^−4^	1.47 × 10^−2^	8.71 × 10^−1^	8.9 × 10^−3^	4.6 × 10^−3^	2.09 × 10^−2^	7.65 × 10^−1^
RBF	6.2 × 10^−3^	2.1 × 10^−3^	1.71 × 10^−2^	7.67 × 10^−1^	1.1 × 10^−2^	5.7 × 10^−3^	2.32 × 10^−2^	7.21 × 10^−1^

**Table 7:** Prediction comparison(mm).

Model	BP	SVM	RF	RBF
Simulated wall thickness results	1.611			
Model prediction result	1.601	1.606	1.598	1.587

**Table 8:** Parameter evaluation.

Model	Training Set				Test Set			
	*MAE*	*MSE*	*RMSE*	*R* ^2^	*MAE*	*MSE*	*RMSE*	*R* ^2^
SVM	3.6 × 10^−3^	8.7 × 10^−8^	1.21 × 10^−3^	9.29 × 10^−1^	5.9 × 10^−3^	2.6 × 10^−3^	1.82 × 10^−2^	9.12 × 10^−1^
SVM-PSO	2.0 × 10^−3^	3.1 × 10^−8^	1.08 × 10^−3^	9.64 × 10^−1^	3.4 × 10^−3^	1.1 × 10^−3^	1.45 × 10^−2^	9.49 × 10^−1^

**Table 9:** Comparison of predicted results with actual production results (mm).

Model	BP	SVM	RF	RBF	PSO–SVM
Production measurement result	1.598				
Forecast result	1.590	1.592	1.587	1.581	1.595
Prediction bias	0.008	0.006	0.011	0.017	0.003

## Data Availability

The original contributions presented in the study are included in the article, further inquiries can be directed to the corresponding author.
